# Development and validation of a clinical predictive model for severe and critical pediatric COVID-19 infection

**DOI:** 10.1371/journal.pone.0275761

**Published:** 2022-10-27

**Authors:** Judith Ju Ming Wong, Qalab Abbas, Felix Liauw, Ririe Fachrina Malisie, Chin Seng Gan, Muhammad Abid, Pustika Efar, Josephine Gloriana, Soo Lin Chuah, Rehena Sultana, Koh Cheng Thoon, Chee Fu Yung, Jan Hau Lee, PACCOVRA Investigators of the PACCMAN research group

**Affiliations:** 1 Department of Pediatric Subspecialties, Children’s Intensive Care Unit, KK Women’s and Children’s Hospital, Singapore, Singapore; 2 SingHealth Duke-NUS Global Health Institute, Singapore, Singapore; 3 Department of Pediatrics and Child Health, Aga Khan University, Karachi, Pakistan; 4 Division of Pediatric Emergency and Intensive Care, Harapan Kita Women and Children Hospital, Jakarta, Indonesia; 5 Child Health Department, Medical Faculty of Universitas Sumatera Utara, Kota Medan, Sumatera Utara, Indonesia; 6 Murni Teguh Memorial Hospital, Kota Medan, Sumatera Utara, Indonesia; 7 Pediatric Intensive Care Unit, Department of Pediatrics, University Malaya Medical Centre, University of Malaya, Kuala Lumpur, Malaysia; 8 Center for Quantitative Medicine, Duke-NUS Medical School, Singapore, Singapore; 9 Department of Pediatrics, Infectious Disease Service, KK Women’s and Children’s Hospital, Singapore, Singapore; 10 Lee Kong Chian School of Medicine, Nanyang Technological University, Singapore, Singapore; 11 Duke-NUS Medical School, Singapore, Singapore; Stellenbosch University Faculty of Medicine and Health Sciences, SOUTH AFRICA

## Abstract

**Introduction:**

Children infected with COVID-19 are susceptible to severe manifestations. We aimed to develop and validate a predictive model for severe/ critical pediatric COVID-19 infection utilizing routinely available hospital level data to ascertain the likelihood of developing severe manifestations.

**Methods:**

The predictive model was based on an analysis of registry data from COVID-19 positive patients admitted to five tertiary pediatric hospitals across Asia [Singapore, Malaysia, Indonesia (two centers) and Pakistan]. Independent predictors of severe/critical COVID-19 infection were determined using multivariable logistic regression. A training cohort (n = 802, 70%) was used to develop the prediction model which was then validated in a test cohort (n = 345, 30%). The discriminative ability and performance of this model was assessed by calculating the Area Under the Curve (AUC) and 95% confidence interval (CI) from final Receiver Operating Characteristics Curve (ROC).

**Results:**

A total of 1147 patients were included in this analysis. In the multivariable model, infant age group, presence of comorbidities, fever, vomiting, seizures and higher absolute neutrophil count were associated with an increased risk of developing severe/critical COVID-19 infection. The presence of coryza at presentation, higher hemoglobin and platelet count were associated with a decreased risk of severe/critical COVID-19 infection. The AUC (95%CI) generated for this model from the training and validation cohort were 0.96 (0.94, 0.98) and 0.92 (0.86, 0.97), respectively.

**Conclusion:**

This predictive model using clinical history and commonly used laboratory values was valuable in estimating the risk of developing a severe/critical COVID-19 infection in hospitalized children. Further validation is needed to provide more insights into its utility in clinical practice.

## Introduction

Since the beginning of COVID-19 pandemic in late 2019, the infection rate and overall disease severity was reported to be low in children [1,2]. Up to January 2022, in the United States, more than 8.5 million children have been infected with an estimated hospitalization rate of 1.7% - 4.3% and a low mortality rate of 0.02% [3]. However, reports of children developing severe COVID-19 infection requiring hospitalization and pediatric intensive care unit (PICU) care are increasing worldwide [4–7]. Children infected with COVID-19 are susceptible to serious manifestations such as acute respiratory distress syndrome (ARDS), shock, stroke, and multisystem inflammatory syndrome in children (MIS-C) [5,8–11]. The reported mortality of these severe phenotypes ranges from 3.5% - 7% [7,12].

Many countries have approved the use of COVID-19 vaccines for children above 12 years of age [13,14] and clinical trials are ongoing to assess its safety and efficacy in adolescents and even younger children (NCT04796896) [15]. However, until these vaccines are approved and made readily available for younger children, they will continue to be at risk of severe infection. Additionally, infants and children with underlying comorbid conditions have been found to be at increased risk for developing severe manifestations of COVID-19 infection [16–18].

In Asia, children may be especially prone to develop severe COVID-19 infection and associated mortality. This susceptibility to severe disease and high mortality rate may be a reflection of socioeconomic factors, cultural factors, hospital admission criteria, management factors and low vaccine coverage [5]. In previous studies from Pakistan and India, children hospitalized with COVID-19 infection or MIS-C had a high mortality (10–20%) [19,20]. Literature also reported various risk factors found to be associated with severe COVID-19 infection and mortality in children; these included: age less than one year, associated comorbid conditions, evidence of acute inflammation and presence of organ dysfunction [21–23]. An early predictive model to identify patients who may progress to severe COVID-19 infection can help stratify patients who may benefit from closer monitoring and admission to a higher level of care. In this study, we aimed to develop and validate a predictive model for severe/ critical pediatric COVID-19 infection utilizing routinely available hospital level data.

## Materials and methods

### Study design

We developed and validated a prediction model based on an analysis of registry data of patients admitted to five tertiary pediatric hospitals across Asia [Singapore, Malaysia, Indonesia (two centers) and Pakistan]. These were centers contributing data to the Pediatric Acute and Critical Care COVID-19 Registry of Asia (PACCOVRA), which is a registry (clinicaltrial.gov registration NCT04395781) within the Pediatric Acute and Critical Care Medicine Asian Network (PACCMAN). This study was approved by the respective institutional ethics boards [Singhealth Centralized Institutional Review Board (2020–2873), Ethics Review Committee, Aga Khan University (2020-4987-11134), Institutional Review Board, Harapan Kita Women’s and Children’s Hospital (IRB/45/11/ETIK/202), Persetujuan Komite Etik Pelaksanaan Penelitian Kesehatan (1173/KEP/USU/2021) and Medical Research Ethics Committee, University Malaya Medical Centre (2020527–8682)] and informed consent was waived at all centers.

Inclusion criteria for patient data were (1) confirmed COVID-19 infection defined by a positive nasopharyngeal aspirate for COVID-19 nucleic acid reverse transcriptase polymerase chain reaction (RT-PCR); or (2) confirmed MIS-C defined by the Centers for Disease Control and Prevention (CDC) criteria [24]; and (3) admitted to participating hospitals from November 2019 to November 2021. Epidemiological, clinical, laboratory and outcome data were extracted retrospectively at participating sites and anonymized data was entered into a secure centralized database set up using Research Electronic Data Capture system (REDCAP) by the main coordinating center in Singapore [25]. Outcome data was captured upon discharge from the hospital. The primary outcome was severe/ critical COVID-19 infection. Severity of COVID-19 infection was classified into four groups based on the World Health Organization (WHO) definition (mild, moderate, severe and critical) [26]. Secondary outcomes included hospital length of stay, final respiratory related diagnosis, respiratory support, supportive therapies and organ dysfunction. Organ dysfunction was defined by the International Pediatric Sepsis Consensus Conference criteria [27]. The management of patients with COVID-19 infection at each participating site was at the discretion of the managing team and no standardised protocol was utilised. The criteria for admission to intermediate care and intensive care was also at the discretion of the managing team.

### Statistical analysis

Primary outcome, COVID-19 infection severity, was categorized as binary data with categories mild/ moderate or severe/ critical infection. All variables were summarized based on COVID-19 infection severity. Categorical and continuous variables were presented as counts (percentages) and median (interquartile range (IQR)), respectively. Chi-Square test and the Mann-Whitney U tests were used to compare categorical and continuous variables, respectively, with respect to COVID-19 infection severity.

The eligible sample (n = 1147) was randomly split into a training (n = 802, 70%) and a validation cohort (n = 345, 30%). Data was randomly split to avoid selection bias for any of the variables in training and validation cohort. The prediction risk model was created using the training cohort. Univariate and multivariable logistic regression model were used to find independent predictors of severe/critical COVID-19 infection. Generalized linear mixed model (GLIMMIX) approach for binary data with site as random effects was used for regression analysis. Covariates considered for inclusion in the model were identified *a priori* without knowledge of the outcome data based on clinical judgement and potential confounders identified in the univariate logistic regression. Variables with p value < 0.2 in the univariate logistic regression model were chosen for multivariable model. Backward, forward and stepwise variable selection were used to determine final predictors of severe/critical COVID-19 infection. The adjusted β coefficient with standard error (SE) and corresponding odds ratio (OR) with 95% confidence intervals (CI) were reported for each predictor. The model constant and β coefficient for each predictor were used to generate the predicted probability equation. The prediction model was assessed by the calibration plot.

The discriminative ability and performance of the model was assessed by calculating the Area Under the Curve (AUC) from final Receiver Operating Characteristics Curve (ROC). Laboratory tests results were incorporated in order to assist clinicians in identifying patients who may develop severe/critical COVID-19 infections. The logistic regression model yields a score based on linear combination of the selected variables. These scores were also reported for full and reduced set of variables. This score can be converted to an estimated probability of severe/critical COVID-19 infection using the relationship: estimated probability = e^score^/ (1+e^score^), where e is the natural exponential. Because laboratory tests (e.g., C-reactive protein, pro-calcitonin) were not mandatory for each center, we expected missing data in these values. For patients who had no admission laboratory data, the first available laboratory data within that admission were used. In our sensitivity analysis, we applied the final multivariable model in two randomly selected sites (KKH and UMMC and again with AKUH and MTMH), and a separate analysis excluding MIS-C patients from both training and validation data to check robustness of the model.

All tests were two sided and statistical significance was set at p value <0.05 unless otherwise stated. Analysis was conducted in R (R Core Team, 2020) and SAS version 9.4 software (SAS Institute; Cary, North Carolina, USA).

## Results

### Study population

**Fig 1:**
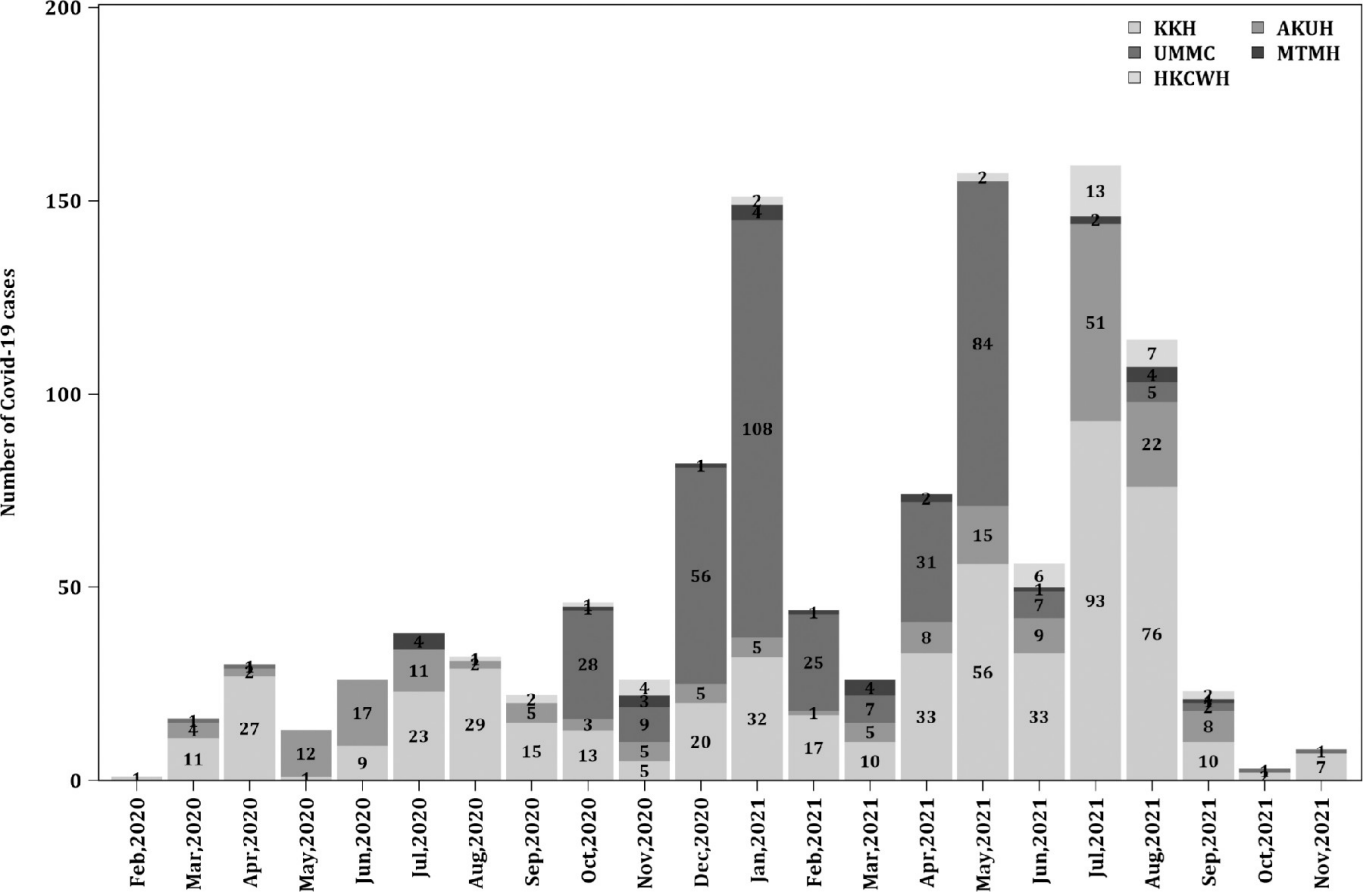
Stacked bar chart of COVID-19 cases from respective sites over the duration of the pandemic. KKH–KK Women’s and Children’s Hospital. UMMC–University Malaya Medical Center. HKCWH–Harapan Kita Women’s and Children’s Hospital. AKUH–Aga Khan University Hospital. MTMH–Murni Teguh Memorial Hospital.

A total of 1147 patients met inclusion criteria for this analysis. The epidemic curve of the pandemic in children from the participating sites is shown in Fig 1. The median (IQR) age was 6 (2, 11) years (Table 1). 982/1147 (85.6%) and 165/1147 (14.4%) patients were in the mild/ moderate and severe/ critical categories, respectively (S1 Table). The most common respiratory diagnosis was upper respiratory tract infection 98/1147 (8.5%) (Tables 2 and S2). Pneumonia and ARDS occurred in 78/1147 (6.8%) and 24/1147 (2.1%) patients respectively, which accounted for the majority of the severe/critical group (S2 Table). Oxygen supplementation, non-invasive ventilation and invasive ventilation was required in 84/1147 (9.5%), 40/1147 (3.5%) and 25/1147 (2.8%) patients, respectively. Ninety-eight of 165 (59.4%) patients in the severe/critical group received systemic corticosteroids, whereas, only 29/165 (17.6%) received intravenous immunoglobulin (IVIG). Empirical use of antibiotics was common especially in the severe/ critical group [151/165 (91.5%)]. Cardiovascular dysfunction occurred in 79/1147 (6.9%) of patients and 58/1147 (5.1%) required vasoactive agents–of these, 14/79 (17.7%) had cardiovascular comorbidities. Respiratory dysfunction occurred in 94/1147 (8.2%)–of these, 9/94 (9.6%) had respiratory comorbidities. MIS-C was diagnosed in 36/1147 (4.3%) of patients. One hundred fifteen of 1147 (12.2%) patients required ICU admission and 33/1147 (2.9%) died–of these, 76/118 (64.4%) had underlying comorbidities.

**Table 1:** Demographic, clinical and laboratory data for the patients in the training and validation dataset.

Variables	Total (n = 1147)	Training dataset (n = 802)	Validation dataset (n = 345)	P value
**Demographic**				
Age, years	6 (2 to 11)	6 (2 to 11)	6 (2 to 11)	0.397
Infant	117 (10.2)	84 (10.5)	33 (9.6)	0.641
Male gender	622 (54.2)	418 (52.1)	204 (59.1)	0.029
Weight, kg	19.4 (10.1 to 35.6)	19.4 (10 to 35)	20 (10.6 to 36.6)	0.458
Appropriate development	844 (92.2)	601 (93.2)	243 (90.0)	0.101
Comorbidity				0.218
Cardiovascular	21 (1.8)	18 (2.2)	3 (0.9)	
Respiratory	42 (3.7)	31 (3.9)	11 (3.2)	
Gastrointestinal	12 (1.0)	9 (1.1)	3 (0.9)	
Hematology oncology	37 (3.2)	28 (3.5)	9 (2.6)	
Neurology	37 (3.2)	23 (2.9)	14 (4.1)	
Others	44 (3.8)	32 (4.0)	12 (3.5)	
**Clinical**				
Fever	527 (45.9)	371 (46.3)	156 (45.2)	0.745
Cough	266 (23.2)	182 (22.7)	84 (24.3)	0.543
Coryza	183 (16.0)	130 (16.2)	53 (15.4)	0.719
Sore throat	109 (9.5)	79 (9.9)	30 (8.7)	0.541
Cyanosis	28 (2.4)	20 (2.5)	8 (2.3)	0.860
Wheezing	29 (2.5)	19 (2.4)	10 (2.9)	0.600
Crepitations	3 (0.3)	2 (0.2)	1 (0.3)	0.902
Other respiratory symptoms	73 (6.4)	44 (5.5)	29 (8.4)	0.063
Headache	38 (3.3)	23 (2.9)	15 (4.3)	0.199
Myalgia	14 (1.2)	12 (1.5)	2 (0.6)	0.195
Irritable	13 (1.1)	8 (1.0)	5 (1.4)	0.507
Feed refusal	17 (1.5)	12 (1.5)	5 (1.4)	0.952
Diarrhea	85 (7.4)	50 (6.2)	35 (10.1)	0.020
Vomiting	97 (8.5)	69 (8.6)	28 (8.1)	0.786
Impaired consciousness	23 (2.0)	17 (2.1)	6 (1.7)	0.673
Seizures	26 (2.3)	20 (2.5)	6 (1.7)	0.431
Others non-respiratory symptoms	143 (12.5)	94 (11.7)	49 (14.2)	0.243
**Laboratory**				
Hemoglobin, g/dL	12.8 (11.9 to 13.7)	12.8 (11.9 to 13.7)	12.8 (11.5 to 13.7)	0.394
WBC, x10/L	7.2 (5.2 to 9.4)	7.3 (5.2 to 9.8)	7.0 (5.4 to 8.7)	0.217
Lymphocyte, x10(9)/L	3.0 (2.0 to 4.6)	3.1 (2.2 to 4.9)	2.9 (1.8 to 4)	0.004
Neutrophil, x10(9)/L	2.9 (1.9 to 4.5)	2.8 (1.8 to 4.5)	3.1 (2.0 to 4.5)	0.298
Platelets, x10(9)/L	294 (230 to 361)	296 (233 to 369)	284.5 (218 to 348)	0.109
APTT, seconds*	32.3 (26.8 to 38.5)	32.2 (27 to 36.8)	32.4 (24.9 to 42.2)	0.912
PT, seconds*	13.3 (11.9 to 14.7)	13.3 (11.9 to 14.7)	12.7 (11.7 to 14.8)	0.928
INR*	1.1 (1 to 1.3)	1.13 (1 to 1.3)	1.1 (1.07 to 1.2)	0.944
D-dimer, FEU*	2.3 (0.5 to 4.5)	2.2 (0.5 to 4.3)	2.5 (0.76 to 4.53)	0.636
Total bilirubin, g/dL*	5 (4 to 7)	5 (4 to 7)	5 (4 to 8)	0.428
AST, U/L*	30 (24 to 38)	29.5 (24 to 38)	30 (23 to 37)	0.534
ALT, U/L*	18 (13 to 25)	17 (13 to 24)	18 (13 to 27)	0.586
Sodium, mmol/L	139 (136 to 142)	140 (136 to 142)	139 (136.5 to 142)	0.446
Potassium, mmol/L	4 (3.5 to 4.4)	4 (3.5 to 4.4)	3.9 (3.5 to 4.4)	0.324
Creatinine, umol/L*	35.4 (26.5 to 53.0)	35.3 (21 to 50)	40 (26.5 to 56)	0.050
C-reactive protein, mg/L*	9.6 (3.1 to 62.1)	8.9 (3.0 to 57.0)	18.5 (4.8 to 74.2)	0.674
Viral co-infection	14 (1.2)	11 (1.4)	3 (0.9)	0.151
Bacterial co-infection	37 (3.2)	24 (3.0)	13 (3.8)	0.337

*High proportion of missing data and were not considered for inclusion in the predictive model

Chi-Square test and the Mann-Whitney U tests were used to compare categorical [counts (%)] and continuous [median (interquartile range)] variables, respectively.

WBC–white blood cell.

APTT–activated partial thromboplastin time.

PT–prothrombin time.

INR- international normalized ratio.

AST–aspartate aminotransferase.

ALT–alanine aminotransferase.

**Table 2:** Diagnosis, complications, therapies and outcomes for patients in the training and validation dataset.

Variables	Total (n = 1147)	Training dataset (n = 802)	Validation dataset (n = 345)	P value
**Respiratory diagnosis**				
URTI	98 (8.5)	64 (8.0)	34 (9.9)	0.298
Bronchiolitis/bronchitis	5 (0.4)	4 (0.5)	1 (0.3)	0.622
Pneumothorax	7 (0.6)	4 (0.5)	3 (0.9)	0.460
Pleural effusion	22 (1.9)	13 (1.6)	9 (2.6)	0.263
Pneumonitis	5 (0.4)	3 (0.4)	2 (0.6)	0.628
Pneumonia	78 (6.8)	54 (6.7)	24 (7.0)	0.890
ARDS	24 (2.1)	14 (1.7)	10 (2.9)	0.211
**Respiratory support**				
Oxygen	84 (9.5)	55 (8.9)	29 (10.8)	0.391
HFNC	16 (1.8)	11 (1.8)	5 (1.9)	0.943
CPAP	11 (1.2)	9 (1.5)	2 (0.7)	0.374
BiPAP	13 (1.5)	11 (1.8)	2 (0.7)	0.235
Mechanical ventilation	25 (2.8)	13 (2.1)	12 (4.5)	0.053
**Other therapies**				
IVIG	29 (2.5)	17 (2.1)	12 (3.5)	0.179
Systemic corticosteroids	122 (10.6)	80 (10.0)	42 (12.2)	0.268
Antibiotics	242 (21.1)	160 (20.0)	82 (23.8)	0.146
Anti-viral	76 (6.6)	53 (6.6)	23 (6.7)	0.971
Anti-fungal	17 (1.5)	9 (1.1)	8 (2.3)	0.124
Vasoactive drugs	58 (5.1)	38 (4.7)	20 (5.8)	0.453
**Organ dysfunction**				
Cardiovascular	79 (6.9)	50 (6.2)	29 (8.4)	0.183
Respiratory	94 (8.2)	64 (8.0)	30 (8.7)	0.685
Neurological	26 (2.3)	16 (2.0)	10 (2.9)	0.346
Hepatic	8 (0.7)	4 (0.5)	4 (1.2)	0.218
Renal	19 (1.7)	11 (1.4)	8 (2.3)	0.249
Hematological	30 (2.6)	17 (2.1)	13 (3.8)	0.109
**Others**				
MIS-C	36 (4.3)	22 (3.8)	14 (5.5)	0.265
Highest level of inpatient care				0.314
General ward	792 (84.2)	557 (85.2)	235 (81.9)	
Intermediate care	31 (3.3)	19 (2.9)	12 (4.2)	
Intensive care	115 (12.2)	75 (11.5)	40 (13.9)	
Hospital duration, days	7 (3 to 12)	7 (3 to 12)	6 (2 to 12)	0.147
Mortality	33 (2.9)	19 (2.4)	14 (4.1)	0.214

Chi-Square test and the Mann-Whitney U tests were used to compare categorical [counts (%)] and continuous [median (interquartile range)] variables, respectively.

URTI–upper respiratory tract infection.

HFNC–high flow nasal cannula.

CPAP–continuous positive airway pressure.

BiPAP–bilevel positive airway pressure.

IVIG–intravenous immunoglobulins.

MIS-C–multisystemic inflammatory syndrome in children.

### Risk score model development and validation

The training and validation dataset were indifferent in all demographic, clinical, laboratory and outcome data, except for a lower lymphocyte count and presenting symptom of diarrhea (Tables 1 and 2). In the multivariable model, infant age group, presence of comorbidities, seizures, vomiting, fever and higher absolute neutrophil count were associated with an increased risk of developing severe/critical COVID-19 infection. The presence of coryza at presentation, hemoglobin and platelet count was associated with a decreased risk severe/critical COVID-19 infection. The severe/critical COVID-19 infection score based on final multivariable model was as follows (Table 3):

Score = 4.29 + 1.87 (comorbidity) + 2.18 (infant) + 3.34 (seizure) + 1.80 (vomiting) + 1.19 (fever)– 1.77 (coryza) + 0.07 (absolute neutrophil count)– 0.57 (hemoglobin)– 0.01 (platelet count)

NB: The β coefficient for each variable indicates the magnitude of effect, whereas the +/- sign indicates the direction of effect. Absolute neutrophil count and platelet count are expressed in 10^9^/L and haemoglobin in g/dL.

**Table 3:** Final multivariable model predicting severe/ critical COVID-19.

Variables	Adjusted β (SE)	Adjusted OR (95%CI)	P value
Intercept	4.29 (1.48)		
Comorbidities (ref: none)	1.87 (0.46)	6.46 (2.62–15.94)	<0.001
Haemoglobin, g/dL	-0.57 (0.11)	0.57 (0.45–0.70)	<0.001
Infant (ref: not infant)	2.18 (0.58)	8.84 (2.82–27.72)	<0.001
Neutrophil, x10(9)/L	0.07 (0.02)	1.08 (1.04–1.11)	<0.001
Seizures (ref: none)	3.34 (1.54)	28.27 (1.39–575.10)	0.030
Vomiting (ref: none)	1.8 (0.54)	6.02 (2.10–17.27)	<0.001
Platelet, x10(9)/L	-0.01 (0.002)	0.99 (0.99–1.00)	<0.001
Coryza (ref: none)	-1.77 (0.84)	0.17 (0.03–0.89)	0.036
Fever (ref: none)	1.19 (0.52)	3.28 (1.19–9.02)	0.021

SE–standard error.

OR–odds ratio.

CI–confidence interval.

**Fig 2:**
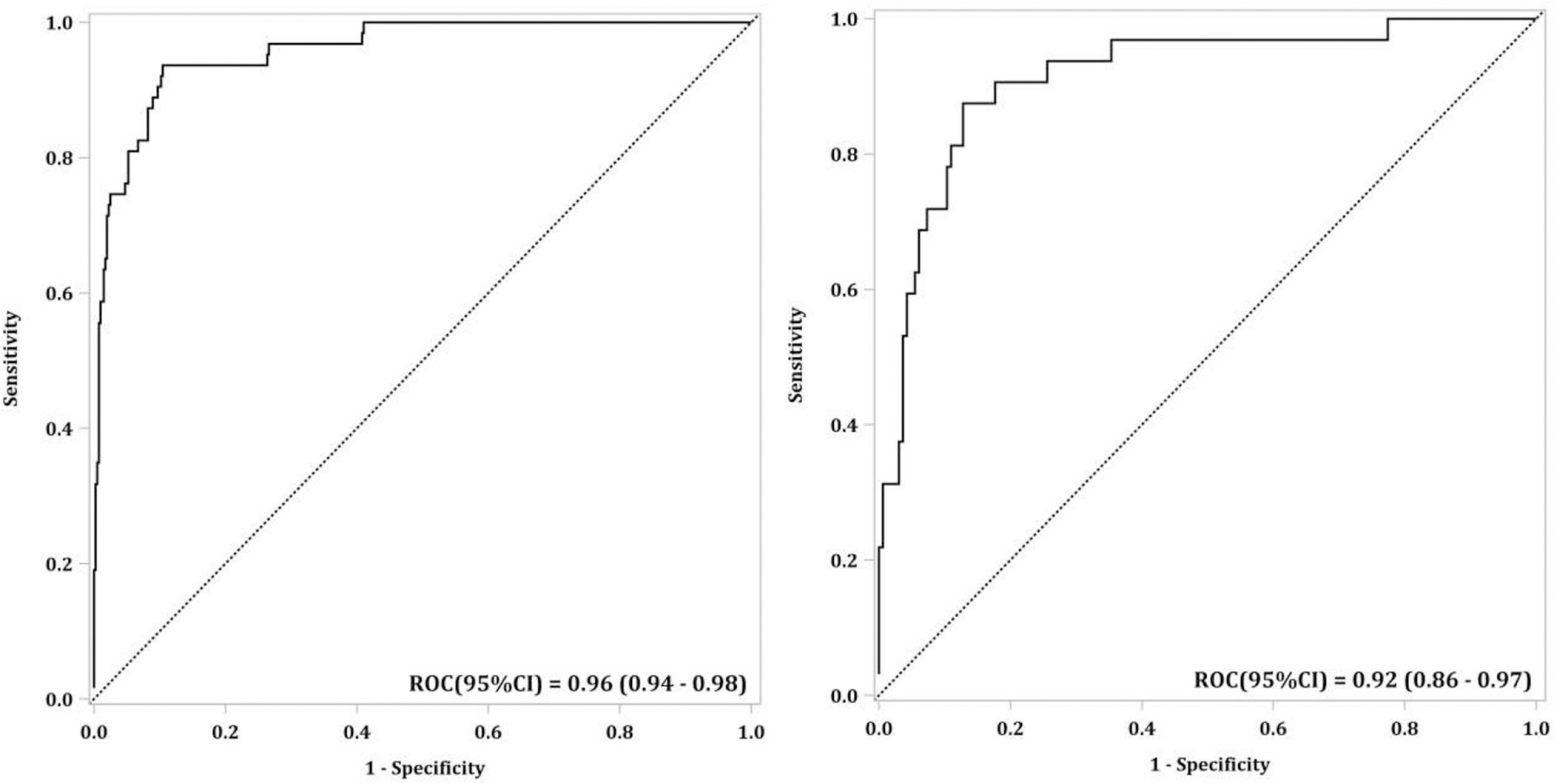
Receiver operating characteristic curve of the training (left) and validation (right) dataset. ROC–Receiver operating characteristic. CI–Confidence interval.

The AUC (95%CI) generated for this model from the training and validation dataset was 0.96 (0.94, 0.98) and 0.92 (0.86, 0.97), respectively (Fig 2). The calibration plot for the training and validation dataset is found in the supplementary material (S1 Fig). This predictive model had a sensitivity and specificity of 53.2% (43.4 to 62.8) and 94.1% (92.1 to 95.7), respectively (S3 Table). In the sensitivity analysis, we applied the predictive model to two random sites to ensure its predictive ability was maintained across sites. The AUCs were satisfactory [0.99 (0.99 to 1.00) and 0.79 (0.71 to 0.86)] (S2 Fig). Calibration plots are provided in the supplementary material (S3 Fig). Excluding the 36 MIS-C patients from analysis also yielded satisfactory training and validation AUCs 0.95 (95%CI 0.92, 0.98) and 0.92 (0.87, 0.96), respectively (S4 Fig).

## Discussion

Utilizing early hospital admission data from a multicenter network, we generated a simple clinical predictive model to identify children who may progress to develop severe/critical COVID-19 disease. Having satisfactory AUC and calibration plots, this model identified the infant age group, presence of comorbidities, fever, vomiting, seizures and a higher absolute neutrophil count to be associated with developing severe/critical COVID-19 disease. The presence of coryza, high hemoglobin and platelet count was, in contrast, protective.

Initial studies have shown that children with COVID-19 infection may not demonstrate the same degree of disease severity compared to adults [11]. However, subsequent studies seem to suggest that there is a bimodal severity peak: The first peak occurs in young infants (<3months) and the second in adulthood. [23,28–30]. Similar to these prior studies conducted in United States and United Kingdom, our predictive model identified infants as a group with increased risk of developing severe COVID-19 disease. The exact reason for this susceptibility remains unclear, though maternal vaccination and breastfeeding practices most likely play a role. The maternal IgG humoral response to vaccination (or infection) has been demonstrated to transfer across the placenta into the fetus, conferring protection to the newborn [31]. Hence, cohorts with a low vaccination rate in expectant mothers (or prior to vaccine approval in pregnancy) may result in inferior protection to the newborn. Although COVID-19 antibodies (SARS-CoV-2 spike RBD-specific IgG1, IgA and IgM antibodies) were detected in breast milk, these were absent in the infants’ serum [32]. On the other hand, other studies, such as a multicenter multivariable Bayesian modeling study conducted in Spain reported that age <2years to be protective against critical COVID-19 infection [33]. The infant age group was therefore included as a discriminatory factor associated with severe COVID-19 infection in our model.

The presence of complex comorbidities increases the risk of hospitalization and severe COVID-19 disease [21,22]. In particular, in a large cross-sectional pediatric study (n = 43,465), cardiovascular diseases, type I diabetes, obesity and prematurity were shown to be associated with severe COVID-19 infection [34]. This is not surprising given that majority of mortalities due to COVID-19 infection occur in patients with underlying metabolic and cardiovascular disease [35]. The predisposition of adult patients with metabolic and cardiovascular comorbidities to COVID-19 disease is recognized to be associated with their pro-inflammatory and hypercoagulopathic tendencies [36]. Obesity also results in altered respiratory mechanics predisposing to severe respiratory infections [37]. It is plausible that these mechanisms also apply to the pediatric patient. Though our study identified the presence of comorbidities to be associated with 6-fold increased odds of severe COVID-19 disease (Table 3), we were not able to replicate with granularity, the contribution of each type of comorbidity due our smaller sample size. Nevertheless, the frequency of cardiovascular, respiratory, gastrointestinal, hematology-oncology and neurology comorbidities were higher in the severe group (S1 Table). The types of hematology-oncology comorbidity and treatments received at the time of study which could have affected outcomes were also not captured.

Symptoms associated with severe COVID-19 infection identified in our study included fever, vomiting and the presence of seizures. Considering that respiratory related COVID-19 disease was more frequent in children [33], it is interesting to note that the presence of non-respiratory symptoms was associated with severe disease. These symptoms potentially indicate systemic involvement. Viral particles have been demonstrated in bronchial and alveolar epithelium, myocardium, intestinal, hepatic, splenic, renal, as well as, brain tissue [38]. Reactive microglia, neuronal ischemia and congestion are among the pathologic findings reported in children with COVID-19 infection who presented with acute encephalopathy and seizures [38]. Our study identifies seizures as a symptom associated with the highest β coefficient for severe disease. It is possible this symptom reflects direct central nervous system (CNS) infection, is part of a systemic syndrome (e.g., shock, cytokine storm, electrolyte imbalance) or part of underlying epilepsy [39]. In contrast, symptoms indicating a mild upper respiratory tract infection (coryza) was evidently protective against severe COVID-19 disease.

Our predictive model included routinely available laboratory variables. Neutrophilia was associated development of severe disease, whereas, higher hemoglobin and platelet were protective. Neutrophilia or relative lymphopenia has been shown to be a feature of both severe respiratory disease and MIS-C [18,19,21,40]. Though less studied, low hemoglobin (anemia) has been associated with an increased risk of respiratory failure, ICU admission, mechanical ventilation and death [41,42]. This hemoglobin effect may be a reflection of disease severity, underlying comorbidity, or malnutrition [42]. Studies have associated thrombocytopenia with critical illness and death in adult COVID-19 infections [43,44]. Interestingly, a trend towards less critical illness was also observed in adult patients with high platelet counts [>400x10^9^/L], though this has not been previously demonstrated in children until our study [43,45]. The mechanism responsible for the protective effect of platelets are unclear but postulated to be related to a protective role of platelets towards the lung parenchyma and improved viral clearance [45].

Though predictive models for severe COVID-19 infection in children have been previously proposed [21,40], these were generated from single countries. Our study utilized data from a network of hospitals across Asia including a population of children with diverse socioeconomic, cultural and biological background which may increase its generalizability. We used routinely available demographic, clinical and laboratory data which is likely relevant in most pediatric admissions to generate the model. However, there were several limitations in this study. Firstly, the diagnosis of MIS-C which requires a recent laboratory confirmed SARS-CoV-2 infection within the prior 4 weeks was challenging in regions where routine PCR or serological evidence of previous infection was not available for patients who had mild symptoms/ asymptomatic primary infections. Some cases may have been missed due to this criteria. Conversely, clinical MIS-C features (e.g. fever, gastro-intestinal symptoms, hypotension and high inflammatory markers) may also be present in acute COVID-19 infection [24,46]. As such, even though the immune-pathology associated with sub-acute/post-acute MIS-C is unique from the cytokine storm of acute COVID-19 infection, the clinical presentation may not be easy to differentiate [47]. We excluded all patients who fulfilled MIS-C criteria in our sensitivity analysis to ensure the predictive model performed satisfactorily regardless of this challenging diagnosis. This limitation also precluded analysis to differentiate patients with/ without COVID-19 immunity. Secondly, we were not able to account for circulating variants of concern which were dominant at the different time periods at each of the sites. For example, the Delta variant (B.1.617.2) of SARS-CoV-2 was dominant in Singapore by May 31^st^ 2021, whereas in Indonesia and Malaysia, it became dominant by July 8^th^ and 23^rd^ 2021, respectively [48–50]. Moreover, availability of sequencing data in some regions was limited and may be subject to sampling bias [51]. Due to the retrospective nature of data collection, the perceived severity of disease may be biased and there was no standardized reporting, laboratory testing or management protocol. As such, we could not investigate other commonly used laboratory tests such as C-reactive protein, procalcitonin and lactate dehydrogenase in the model [52]. Further external validation is needed to evaluate the performance of this predictive model in the clinical setting and in other geographical regions. Lastly, we did not explore other important outcomes including mortality (due to the small sample size, n = 33) and the cause of mortality reported in this study may or may not be directly due to COVID-19 infection.

## Conclusion

In summary, we created a predictive model to identify children who may develop severe/critical COVID-19 infection using routinely available hospital level data. This novel model should be validated further in other settings and potentially useful to hospitalists in helping stratify patients into those may benefit from closer monitoring in a higher level of care.

## Supporting information

S1 Fig Calibration plots for the training (left) and validation (right) dataset.
(TIF)

S2 Fig Receiver operating characteristic curve for two randomly selected sites KK Women’s and Children’s Hospital and University Malaya Medical Center (left), and Aga Khan University Hospital and Murni Teguh Memorial Hospital (right).
(TIF)

S3 Fig Calibration plots for two randomly selected sites KK Women’s and Children’s Hospital and University Malaya Medical Center (left), and Aga Khan University Hospital and Murni Teguh Memorial Hospital (right).
(TIF)

S4 Fig Receiver operating characteristic curve excluding patients fulfilling MISC criteria. (TIF)

S1 Table Demographic, clinical and laboratory data for the patients in the mild/moderate and severe/critical COVID-19 group. (DOCX)

S2 Table Diagnosis, complications, therapies and outcomes data for the patients in the mild/moderate and severe/critical COVID-19 group. (DOCX)

S3 Table Diagnostic accuracy of the predictive model for severe/critical COVID-19. (DOCX)

## Abbreviations

AUC: Area under the curve
COVID-19: Coronavirus disease 2019
CI: confidence interval
MIS-C: Multisystem inflammatory syndrome in children
ROC: Receiver operating characteristics
SARS-CoV-2: Severe acute respiratory syndrome coronavirus 2
