# Supplementary figures and images for: Development and validation of a clinical predictive model for severe and critical pediatric COVID-19 infection
Source: PLoS One. 2022 Oct 27;17(10):e0275761. doi: 10.1371/journal.pone.0275761 (PMC9612577; doi:10.1371/journal.pone.0275761)

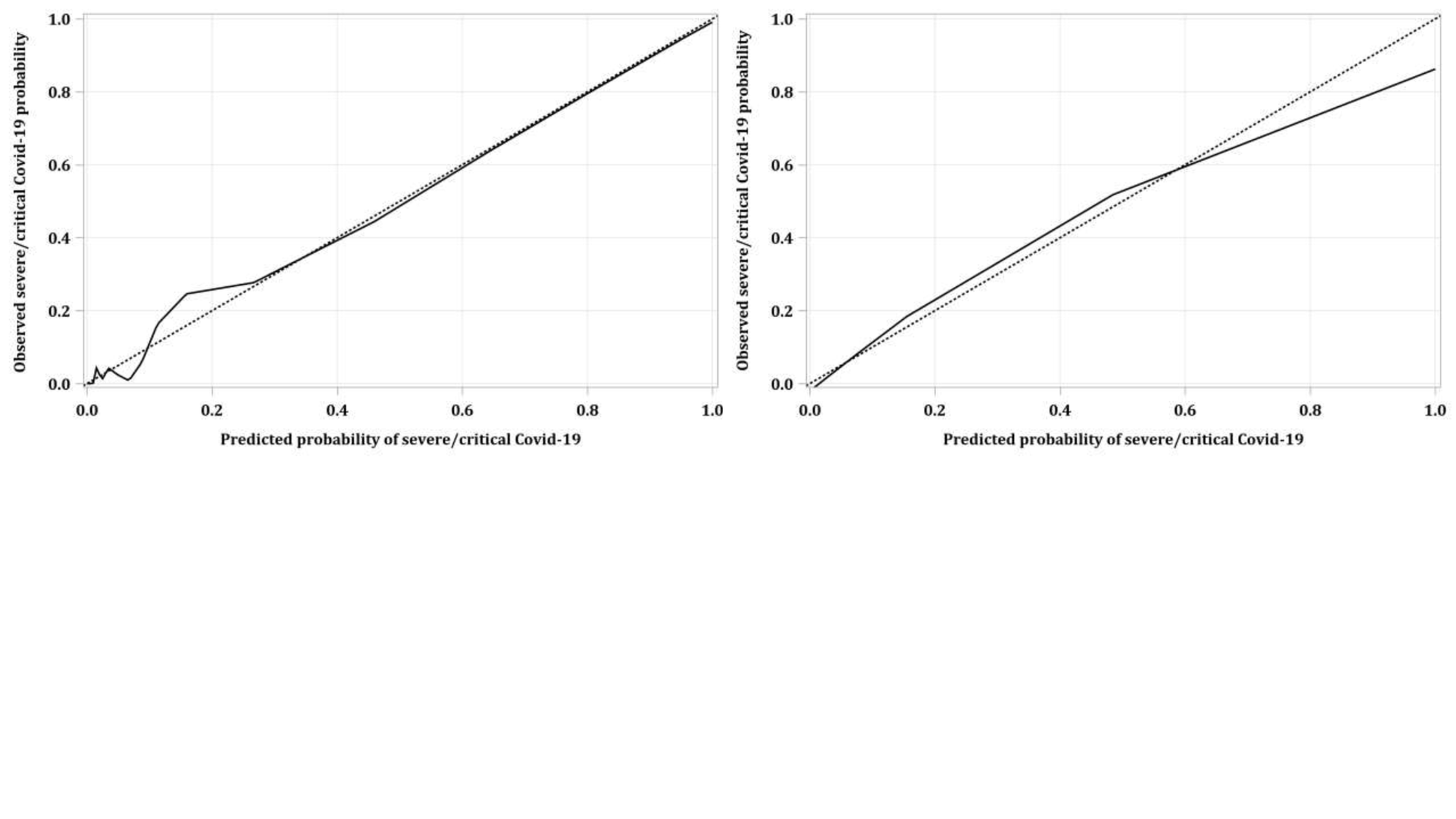

Supplement: S1 Fig — Calibration plots for the training (left) and validation (right) dataset. (TIF) [file pone.0275761.s001.tif]

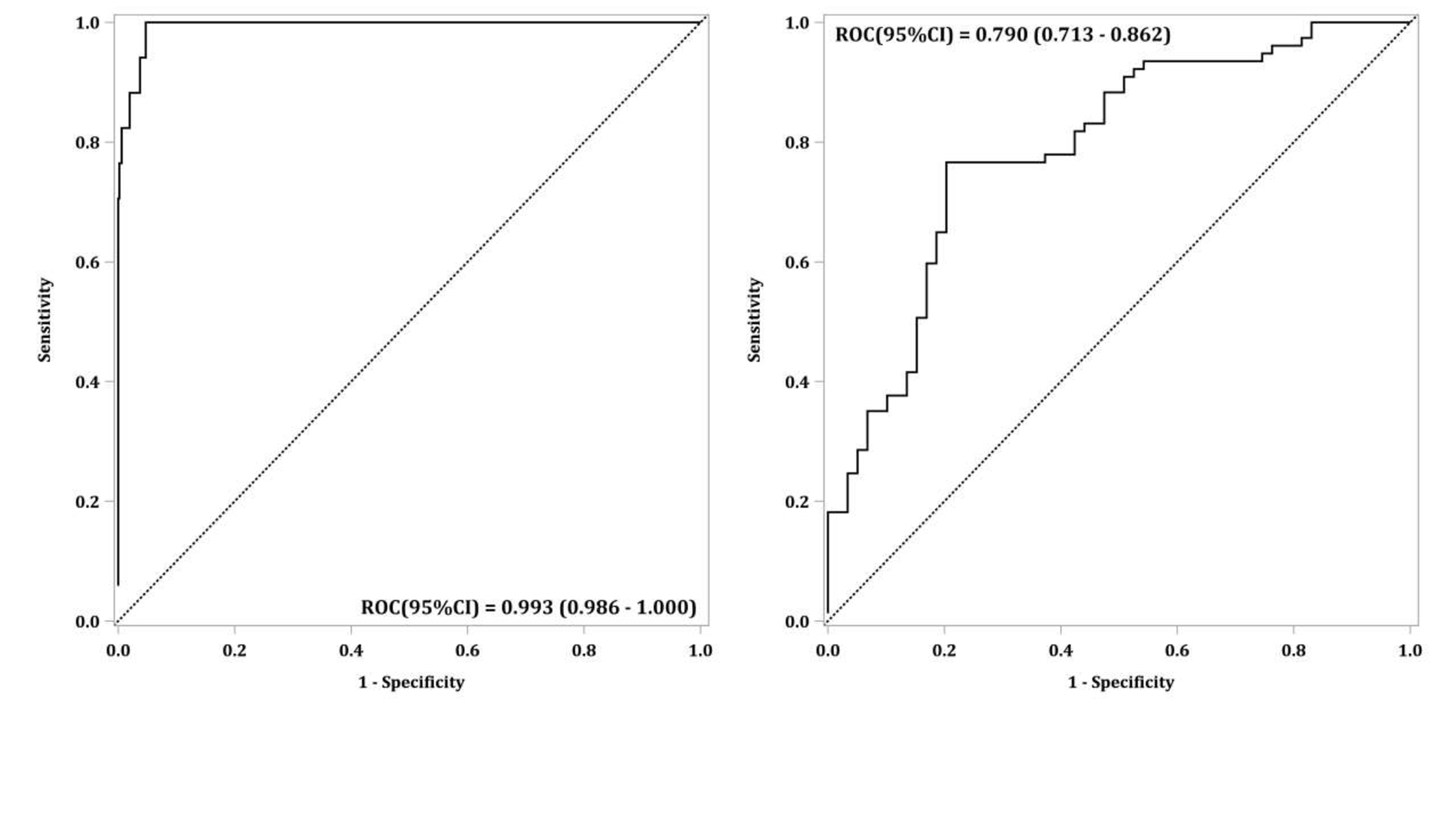

Supplement: S2 Fig — Receiver operating characteristic curve for two randomly selected sites KK Women’s and Children’s Hospital and University Malaya Medical Center (left), and Aga Khan University Hospital and Murni Teguh Memorial Hospital (right). (TIF) [file pone.0275761.s002.tif]

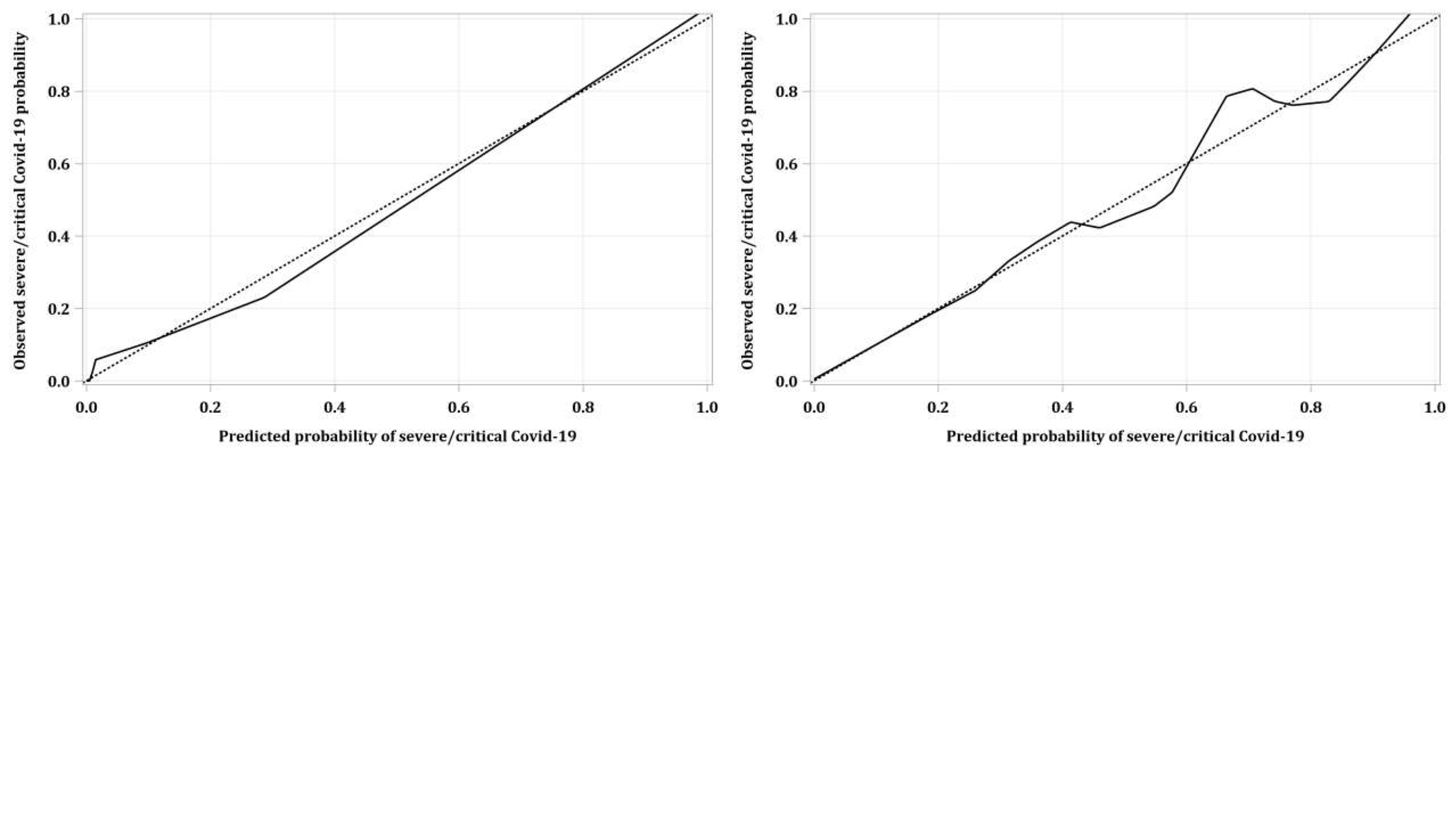

Supplement: S3 Fig — Calibration plots for two randomly selected sites KK Women’s and Children’s Hospital and University Malaya Medical Center (left), and Aga Khan University Hospital and Murni Teguh Memorial Hospital (right). (TIF) [file pone.0275761.s003.tif]

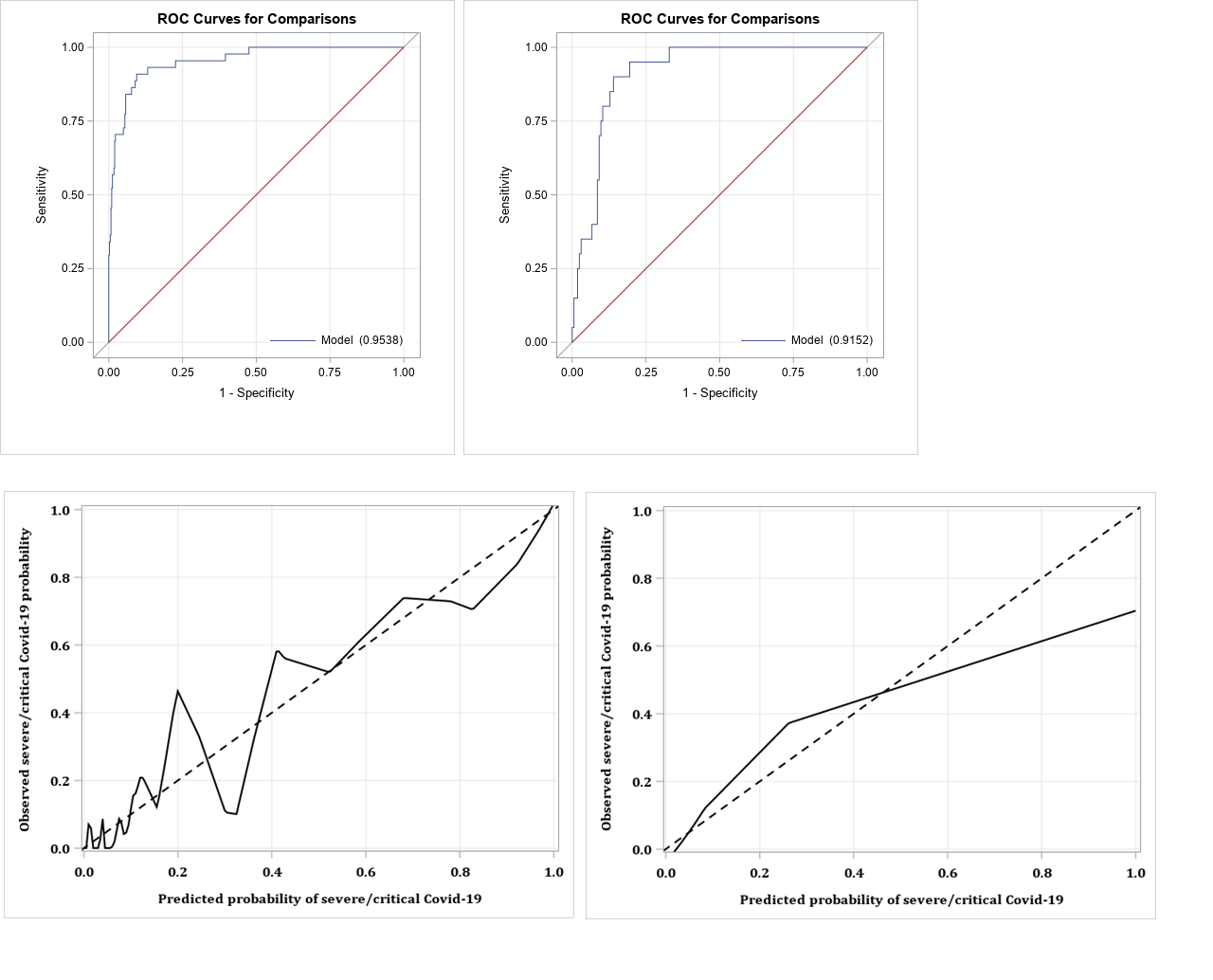

Supplement: S4 Fig — (TIF) [file pone.0275761.s004.tif]
