# Supplementary material for: Development and validation of a clinical predictive model for severe and critical pediatric COVID-19 infection
Source: PLoS One. 2022 Oct 27;17(10):e0275761. doi: 10.1371/journal.pone.0275761 (PMC9612577; doi:10.1371/journal.pone.0275761)
Supplement: S1 Table — (DOCX) [file pone.0275761.s005.docx]

**S1 Table: Demographic, clinical and laboratory data for the patients in the mild/moderate and severe/critical COVID-19 group**

|  | **Training dataset** | | | | **Validation dataset** | | | |
| --- | --- | --- | --- | --- | --- | --- | --- | --- |
| **Variable** | **Total**  **(n=802)** | **Mild/ moderate (n=693)** | **Severe/ critical (n=109)** | **P value** | **Total**  **(n=345)** | **Mild/ moderate (n=289)** | **Severe/ critical (n=56)** | **P value** |
| Age, years | 6 (2 to 11) | 7 (3 to 10) | 4 (1 to 12) | 0.076 | 6 (2 to 11) | 6 (3 to 12) | 4 (1 to 11) | 0.084 |
| Infant | 84 (10.5) | 56 (8.1) | 28 (25.7) | <0.001 | 33 (9.6) | 20 (6.9) | 13 (23.2) | <0.001 |
| Male gender | 418 (52.1) | 350 (50.5) | 68 (62.4) | 0.021 | 204 (59.1) | 171 (59.2) | 33 (58.9) | 0.973 |
| Weight, kg | 19.4 (10 to 35) | 19.8 (11 to 35) | 15 (6.7 to 37.8) | 0.116 | 20 (10.6 to 36.6) | 20 (10.7 to 37.8) | 17.9 (9.6 to 34.4) | 0.775 |
| Appropriate development | 601 (93.2) | 529 (97.4) | 72 (70.6) | <0.001 | 243 (90.0) | 204 (94.0) | 39 (73.6) | <0.001 |
| Comorbidity |  |  |  | <0.001 |  |  |  | <0.001 |
| Cardiovascular | 18 (2.2) | 4 (0.6) | 14 (13.0) |  | 3 (0.9) | 1 (0.3) | 2 (3.6) |  |
| Respiratory | 31 (3.9) | 21 (3.0) | 10 (9.3) |  | 11 (3.2) | 5 (1.7) | 6 (10.7) |  |
| Gastrointestinal | 9 (1.1) | 6 (0.9) | 3 (2.8) |  | 3 (0.9) | 1 (0.3) | 2 (3.6) |  |
| Hematology oncology | 28 (3.5) | 11 (1.6) | 17 (15.7) |  | 4 (1.4) | 4 (1.4) | 5 (8.9) |  |
| Neurology | 23 (2.9) | 7 (1.0) | 16 (14.8) |  | 6 (2.1) | 6 (2.1) | 8 (14.3) |  |
| Others | 32 (4.0) | 22 (3.2) | 10 (9.3) |  | 6 (2.1) | 6 (2.1) | 6 (10.7) |  |
| **Clinical** |  |  |  |  |  |  |  |  |
| Fever | 371 (46.3) | 291 (42.0) | 80 (73.4) | <0.001 | 156 (45.2) | 119 (41.2) | 37 (66.1) | <0.001 |
| Cough | 182 (22.7) | 146 (21.1) | 36 (33.0) | 0.006 | 84 (24.3) | 62 (21.5) | 22 (39.3) | 0.004 |
| Coryza | 130 (16.2) | 125 (18.0) | 5 (4.6) | <0.001 | 53 (15.4) | 52 (18.0) | 1 (1.8) | 0.002 |
| Sore throat | 79 (9.9) | 79 (11.4) | 0 (0.0) | <0.001 | 30 (8.7) | 30 (10.4) | 0 (0.0) | 0.012 |
| Cyanosis | 20 (2.5) | 1 (0.1) | 19 (17.4) | <0.001 | 8 (2.3) | 0 (0.0) | 8 (14.3) | <0.001 |
| Wheezing | 19 (2.4) | 1 (0.1) | 18 (16.5) | <0.001 | 10 (2.9) | 0 (0.0) | 10 (17.9) | <0.001 |
| Crepitations | 2 (0.2) | 0 (0.0) | 2 (1.8) | <0.001 | 1 (00.3) | 0 (0.0) | 1 (1.8) | 0.023 |
| Other respiratory symptoms | 44 (5.5) | 17 (2.5) | 27 (24.8) | <0.001 | 29 (8.4) | 13 (4.5) | 16 (28.6) |  |
| Headache | 23 (2.9) | 20 (2.9) | 3 (2.8) | 0.938 | 15 (4.3) | 13 (4.5) | 2 (3.6) | 0.756 |
| Myalgia | 12 (1.5) | 10 (1.4) | 2 (1.8) | 0.754 | 2 (0.6) | 2 (0.7) | 0 (0.0) | 0.532 |
| Irritable | 8 (1.0) | 2 (0.3) | 6 (5.5) | <0.001 | 5 (1.4) | 2 (0.7) | 3 (5.4) | 0.008 |
| Feed refusal | 12 (1.5) | 6 (0.9) | 6 (5.5) | <0.001 | 5 (1.4) | 2 (0.7) | 3 (5.4) | 0.008 |
| Diarrhea | 50 (6.2) | 30 (4.3) | 20 (18.3) | <0.001 | 35 (10.1) | 23 (8.0) | 12 (21.4) | 0.002 |
| Vomiting | 69 (8.6) | 38 (5.5) | 31 (28.4) | <0.001 | 28 (8.1) | 14 (4.8) | 14 (25.0) | <0.001 |
| **Laboratory** |  |  |  |  |  |  |  |  |
| Hemoglobin, g/dL | 12.8 (11.9 to 13.7) | 13.0 (12.3 to 13.8) | 10.2 (8.7 to 12.0) | <0.001 | 12.8 (11.5 to 13.7) | 13 (12.1 to 13.7) | 10.1 (8.8 to 12.1) | <0.001 |
| WBC, x10/L | 7.3 (5.2 to 9.8) | 7.1 (5.2 to 9.4) | 9.4 (5.4 to 14.4) | 0.002 | 7.0 (5.4 to 8.7) | 7.1 (5.5 to 8.6) | 6.6 (4.0 to 9.3) | 0.61 |
| Lymphocyte, x10(9)/L | 3.1 (2.2 to 4.9) | 3.2 (2.3 to 4.8) | 2.6 (1.2 to 5.5) | 0.026 | 2.9 (1.8 to 4.0) | 3.0 (2.1 to 4.5) | 1.1 (0.5 to 2.0) | <0.001 |
| Neutrophil, x10(9)/L | 2.8 (1.1 to 4.5) | 2.7 (1.8 to 4.0) | 6.0 (3.1 to 14.1) | <0.001 | 3.1 (2.0 to 4.5) | 2.8 (1.9 to 4.2) | 4.7 (3.4 to 7.7) | <0.001 |
| Platelets, x10(9)/L | 296 (233 to 369) | 303 (249 to 371) | 225 (127 to 331) | <0.001 | 284.5 (218 to 348) | 298 (249 to 352) | 194 (67.5 to 263) | <0.001 |
| APTT, seconds* | 32.2 (27.0 to 36.8) | 30.4 (24.5 to 32.5) | 32.8 (28.1 to 40.8) | 0.057 | 32.4 (24.9 to 42.2) | 29.8 (25.4 to 34.7) | 33.1 (23.7 to 44.7) | 0.477 |
| PT, seconds* | 13.3 (11.9 to 14.7) | 13.0 (12.2 to 14.0) | 13.4 (11.9 to 15.1) | 0.307 | 12.7 (11.7 to 14.8) | 13.5 (11.1 to 14.5) | 12.7 (12.0 to 15.4) | 0.424 |
| INR* | 1.1 (1.0 to 1.3) | 1 (1.0 to 1.2) | 1.2 (1.1 to 1.4) | 0.002 | 1.1 (1.07 to 1.2) | 1.1 (1 to 1.1) | 1.2 (1.1 to 1.3) | 0.003 |
| D-dimer, FEU* | 2.2 (0.5 to 4.3) | 0.4 (0.3 to 1.2) | 3.0 (1.4 to 5.2) | <0.001 | 2.5 (0.8 to 4.5) | 0.8 (0.3 to 2.9) | 3.15 (1.3 to 9.1) | 0.028 |
| Total bilirubin, g/dL* | 5 (4 to 7) | 5 (4 to 7) | 9.4 (5.5 to 15.3) | <0.001 | 5 (4 to 8) | 5 (4 to 7) | 11.1 (6.8 to 20) | 0.001 |
| AST, U/L* | 29.5 (24.0 to 38.0) | 29 (24 to 37) | 49 (31 to 59) | <0.001 | 30 (23 to 37) | 28 (22 to 36) | 43 (33.5 to 63.5) | <0.001 |
| ALT, U/L* | 17.0 (13.0 to 24.0) | 17 (13 to 23) | 37 (21 to 51) | <0.001 | 18 (13 to 27) | 17 (13 to 23) | 41 (27 to 91) | <0.001 |
| Sodium, mmol/L | 140 (136 to 142) | 140 (138 to 142) | 138 (134 to 143) | 0.139 | 139 (136.5 to 142) | 139 (137 to 141) | 138 (133 to 142) | 0.786 |
| Potassium, mmol/L | 4.0 (3.5 to 4.4) | 4.3 (3.9 to 4.7) | 3.8 (3.2 to 4.4) | <0.001 | 3.9 (3.5 to 4.4) | 4.05 (3.8 to 4.4) | 3.65 (3.3 to 4.4) | 0.029 |
| Creatinine, umol/L* | 35.36 (21 to 50) | 32 (20 to 44.2) | 35.4 (26.5 to 53.0) | 0.203 | 40 (26.5 to 56) | 44.2 (31 to 57) | 35.4 (26.5 to 56) | 0.718 |
| C-reactive protein, mg/L* | 8.9 (3.0 to 57.0) | 3.2 (0.9 to 5) | 36.0 (9.2 to 113.1) | <0.001 | 18.5 (4.8 to 74.2) | 5 (0.4 to 8.4) | 55.7 (22.1 to 132.8) | <0.001 |

*High proportion of missing data and were not considered for inclusion in the predictive model

WBC – white blood cell

APTT – activated partial thromboplastin time

PT – prothrombin time

INR- international normalized ratio

AST – aspartate aminotransferase

ALT – alanine aminotransferase
