# Supplementary material for: Development and validation of a clinical predictive model for severe and critical pediatric COVID-19 infection
Source: PLoS One. 2022 Oct 27;17(10):e0275761. doi: 10.1371/journal.pone.0275761 (PMC9612577; doi:10.1371/journal.pone.0275761)
Supplement: S2 Table — (DOCX) [file pone.0275761.s006.docx]

**S2 Table: Diagnosis, complications, therapies and outcomes data for the patients in the mild/moderate and severe/critical COVID-19 group**

|  | **Training dataset** | | | | **Validation dataset** | | | |
| --- | --- | --- | --- | --- | --- | --- | --- | --- |
| **Variable** | **Total (n=802)** | **Mild/ moderate (n=693)** | **Severe/ critical (n=109)** | **P value** | **Total**  **(n=345)** | **Mild/ moderate (n=289)** | **Severe/ critical (n=56)** | **P value** |
| Viral co-infection | 11 (1.4) | 6 (0.9) | 5 (4.6) | 0.002 | 3 (0.9) | 2 (0.7) | 1 (1.8) | 0.157 |
| Bacterial co-infection | 24 (3.0) | 5 (0.7) | 19 (17.4) | <0.001 | 13 (3.8) | 5 (1.7) | 8 (14.3) | 0.336 |
| Respiratory diagnosis |  |  |  |  |  |  |  |  |
| URTI | 64 (8.0) | 55 (7.9) | 9 (8.3) | 0.909 | 34 (9.9) | 31 (10.7) | 3 (5.4) | 0.217 |
| Bronchiolitis/bronchitis | 4 (0.5) | 02 (0.3) | 2 (1.8) | 0.033 | 1 (0.3) | 1 (0.3) | 0 (0.0) | 0.659 |
| Pneumothorax | 4 (0.5) | 0 (0.0) | 4 (3.7) | <0.001 | 3 (0.9) | 0 (0.0) | 3 (5.4) | <0.001 |
| Pleural effusion | 13 (1.6) | 0 (0.0) | 13 (11.9) | <0.001 | 9 (2.6) | 1 (0.3) | 8 (14.3) | <0.001 |
| Pneumonitis | 3 (0.4) | 0 (0.0) | 3 (2.8) | <0.001 | 2 (0.6) | 0 (0.0) | 2 (3.6) | 0.001 |
| Pneumonia | 54 (6.7) | 3 (0.4) | 51 (46.8) | <0.001 | 24 (7.0) | 0 (0.0) | 24 (42.9) | <0.001 |
| ARDS | 14 (1.7) | 0 (0.0) | 14 (12.8) | <0.001 | 10 (2.9) | 0 (0.0) | 10 (17.9) | <0.001 |
| Respiratory support |  |  |  |  |  |  |  |  |
| Oxygen | 55 (8.9) | 0 (0.0) | 55 (64.7) | <0.001 | 29 (10.8) | 0 (0.0) | 29 (61.7) | <0.001 |
| HFNC | 11 (1.8) | 1 (0.2) | 10 (11.8) | <0.001 | 5 (1.9) | 0 (0.0) | 5 (10.6) | <0.001 |
| CPAP | 9 (1.5) | 0 (0.0) | 9 (10.6) | <0.001 | 2 (0.7) | 0 (0.0) | 2 (4.3) | 0.002 |
| BiPAP | 11 (1.8) | 0 (0.0) | 11 (12.9) | <0.001 | 2 (0.7) | 0 (0.0) | 2 (4.3) | 0.002 |
| Mechanical ventilation | 13 (2.1) | 0 (0.0) | 13 (15.3) | <0.001 | 12 (4.5) | 0 (0.0) | 12 (25.5) | <0.001 |
| Other therapies |  |  |  |  |  |  |  |  |
| IVIG | 17 (2.1) | 0 (0.0) | 17 (15.6) | <0.001 | 12 (3.5) | 0 (0.0) | 12 (21.4) | <0.001 |
| Systemic corticosteroids | 80 (10.0) | 18 (2.6) | 62 (56.9) | <0.001 | 42 (12.2) | 6 (2.1) | 36 (64.3) | <0.001 |
| Antibiotics | 160 (20.0) | 60 (8.7) | 100 (91.7) | <0.001 | 82 (23.8) | 31 (10.7) | 51 (91.1) | <0.001 |
| Anti-viral | 53 (6.6) | 18 (2.6) | 35 (32.1) | <0.001 | 23 (6.7) | 6 (2.1) | 17 (30.4) | <0.001 |
| Anti-fungal | 9 (1.1) | 1 (0.1) | 8 (7.3) | <0.001 | 8 (2.3) | 0 (0.0) | 8 (14.3) | <0.001 |
| Vasoactive drugs | 38 (4.7) | 0 (0.0) | 38 (34.9) | <0.001 | 20 (5.8) | 0 (0.0) | 20 (35.7) | <0.001 |
| Organ dysfunction |  |  |  |  |  |  |  |  |
| Cardiovascular | 50 (6.2) | 0 (0.0) | 50 (45.9) | <0.001 | 29 (8.4) | 0 (0.0) | 29 (51.8) | <0.001 |
| Respiratory | 64 (8.0) | 1 (0.1) | 63 (57.8) | <0.001 | 30 (8.7) | 0 (0.0) | 30 (53.6) | <0.001 |
| Neurological | 16 (2.0) | 0 (0.0) | 16 (14.7) | <0.001 | 10 (2.9) | 0 (0.0) | 10 (17.9) | <0.001 |
| Hepatic | 4 (0.5) | 2 (0.3) | 2 (1.8) | 0.033 | 4 (1.2) | 0 (0.0) | 4 (7.1) | <0.001 |
| Renal | 11 (1.4) | 0 (0.0) | 11 (10.1) | <0.001 | 8 (2.3) | 0 (0.0) | 8 (14.3) | <0.001 |
| Hematological | 17 (2.1) | 1 (0.1) | 16 (14.7) | <0.001 | 13 (3.8) | 1 (0.3) | 12 (21.4) | <0.001 |
| Others |  |  |  |  |  |  |  |  |
| MIS-C | 22 (3.8) | 0 (0.0) | 22 (20.8) | <0.001 | 14 (5.5) | 0 (0.0) | 14 (26.9) | <0.001 |
| Highest level of inpatient care |  |  |  | <0.001 |  |  |  | <0.001 |
| General ward | 557 (85.2) | 538 (98.7) | 19 (17.4) |  | 235 (81.9) | 227 (98.3) | 8 (14.3) |  |
| Intermediate care | 19 (2.9) | 4 (0.7) | 15 (13.8) |  | 12 (4.2) | 1 (0.4) | 11 (19.6) |  |
| Intensive care | 75 (11.5) | 3 (0.6) | 72 (66.1) |  | 40 (13.9) | 3 (1.3) | 37 (66.1) |  |
| Hospital duration, days | 7 (3 to 12) | 7.5 (3 to 12) | 6.5 (4 to 11.5) | 0.992 | 6 (2 to 12) | 6 (2 to 12) | 7 (4 to 14) | 0.172 |
| Mortality | 19 (2.4) | 0 (0.0) | 19 (17.6) | <0.001 | 14 (4.1) | 0 (0.0) | 14 (25.0) | <0.001 |

Chi-Square test and the Mann-Whitney U tests were used to compare categorical [counts (%)] and continuous [median (interquartile range)] variables, respectively.

URTI – upper respiratory tract infection

HFNC – high flow nasal cannula

CPAP – continuous positive airway pressure

BiPAP – bilevel positive airway pressure

IVIG – intravenous immunoglobulins

MIS-C – multisystemic inflammatory syndrome in children
