# Supplementary material for: Development and validation of a clinical predictive model for severe and critical pediatric COVID-19 infection
Source: PLoS One. 2022 Oct 27;17(10):e0275761. doi: 10.1371/journal.pone.0275761 (PMC9612577; doi:10.1371/journal.pone.0275761)
Supplement: S3 Table — (DOCX) [file pone.0275761.s007.docx]

**S3 Table: Diagnostic accuracy of the predictive model for severe/critical COVID-19**

| **Statistic** | **Value** | **95% CI** |
| --- | --- | --- |
| Sensitivity | 53.2% | 43.4 to 62.8% |
| Specificity | 94.1% | 92.1 to 95.7% |
| Positive Likelihood Ratio | 9.0 | 6.4 to 12.7 |
| Negative Likelihood Ratio | 0.5 | 0.4 to 0.6 |
| Disease prevalence | 13.6% | 11.3 to 16.2% |
| Positive Predictive Value | 58.6% | 50.0 to 66.6% |
| Negative Predictive Value | 92.8% | 91.3 to 94.0% |
| Accuracy | 88.5% | 86.1 to 90.7% |
